# Editorial: Teacher Emotions Matter—Nature, Antecedents, and Effects

**DOI:** 10.3389/fpsyg.2020.605389

**Published:** 2020-11-17

**Authors:** Junjun Chen, Hongbiao Yin, Anne C. Frenzel

**Affiliations:** ^1^Department of Education Policy and Leadership, Education University of Hong Kong, Hong Kong, China; ^2^Department of Curriculum and Instruction, Chinese University of Hong Kong, Hong Kong, China; ^3^Department of Psychology, University of Munich, Munich, Germany

**Keywords:** teacher emotion, nature, antecedent, effects, special issue

Along with increasing recognition of the varied aspects of education, research on teacher emotions has blossomed recently after being unacknowledged for decades. The statistics from Scopus indicate that the number of journal articles published on teacher emotions in the past 5 years is 497, comprising the largest proportion (61%) of the 812 total article corpus on the topic over the past 35 years^1^. Despite this notable growth, the field is still in a pre-mature developmental stage as it lacks a full consideration of the complexities of teacher emotions, and a balanced coverage of research foci and methodologies (Frenzel, 2014; Fried et al., 2015; Chen, 2019, 2020; Yin et al., 2019). In particular, Fried et al. (2015) have argued that “the study of teacher emotion is in need of conceptual clarity” (p. 415). Likewise, Chen (2019) identified a clear need for advancing mixed-method and longitudinal studies on the topic as the existing literature on teacher emotions largely relies on self-report data and cross-sectional research designs.

Although interest in the field has been growing since the first special issue by Nias (1996) in the *Cambridge Journal of Education*, teacher emotions have previously been addressed in only one single virtual special issue which focused on articles published in *Teaching and Teacher Education* by Uitto et al. (2015). The present Research Topic in *Frontiers in Psychology* thus aims to provide a platform for showcasing the latest research on teacher emotions, to acknowledge its increasingly important scientific impact.

Inspired by the reciprocal model of teacher emotions proposed by Frenzel (2014), the current Research Topic is entitled “Teacher Emotions Matter: Nature, Antecedents, and Effects.” It was designed to spark the publication of new empirical evidence about potential reciprocal linkages between teacher emotions and other constructs, and thus contribute to the conceptual framework within the teacher emotion literature. Furthermore, this Research Topic sought to embrace a corpus of robust research covering various research foci and innovative methodologies, aiming at maturing the evolving conceptual understanding of teacher emotions. The 16 papers which comprise this Research Topic in many ways achieve this claim, even though again, there is a predominance of cross-sectional designs and self-report-based methods of inquiry. The collection of papers consists of two parts. Part 1 explicitly covers teachers' emotional and affective experiences, with papers one through four addressing the nature of teacher emotions, papers five through seven focusing on antecedents, papers eight and nine focusing on effects, and papers 10 and 11 focusing on reciprocal linkages. Part 2 addresses teachers' emotion regulation and emotional competence (papers 12 through 16). Table 1 provides an overview of all 16 papers' focal emotional variables and their conceptualization, methodological approach, and samples. Below, we highlight key findings from each paper.

**Table 1:** Overview of Research Topic papers.

**References**	**Article title**	**Focal emotional variables and their conceptualization**	**Methodological approach**	**Sample**
**PART A: TEACHERS' EMOTIONAL EXPERIENCES**				
**Section A.1. Nature**				
Chen et al.	A1. Understanding the complexity of teacher emotions from online forums: A computational text analysis approach	Discrete emotions (i.e., anger, anticipation, disgust, fear, joy, sadness, surprise, and trust) and sentiment polarity (i.e., positive, negative, and neutral)	Computational text analysis of online posts	School teachers
Chen et al.	A2. Emotional trajectory at different career stages: Two excellent teachers' stories	“Salient negative and positive emotions” (in the sense of “internalized sensations… integral to the ways in which they relate to and interact with their students, colleagues and parents”) and emotional labor strategies: surface acting, deep acting, and genuine expression	Interviews	School teachers
Cross Francis et al.	A3. The dominance of blended emotions: A qualitative study of elementary teachers' emotions related to mathematics teaching	Emotions as “socially constructed, personally enacted ways of being that emerge from conscious and/or unconscious judgments regarding perceived successes at attaining goals or maintaining standards or beliefs during transactions as part of social-historical contexts”	Interviews	Elementary school teachers
Tsang	A4. The interactional-institutional construction of teachers' emotions in Hong Kong: The inhabited institutionalism perspective	Negative emotions (“such as dissatisfaction, stress, depression, and anxiety”)	Interviews and document analysis	Secondary school teachers
**Section A.2. Focus on Antecedents**				
Yoo and Rho	A5. Exploration of predictors for Korean teacher job satisfaction via a machine learning technique, Group Mnet	Job satisfaction in terms of “the sense of fulfillment and gratification from working in a specific occupation”; specifically, the role and work of a teacher, as well as satisfaction with the school environment.”	Self-report questionnaires (OECD TALIS), cross-sectional design	Middle school teachers
Büssing et al.	A6. Topic specificity and antecedents for pre-service biology teachers' anticipated enjoyment for teaching about socio-scientific issues: Investigating universal values and psychological distance	Anticipated teaching enjoyment	Self-report questionnaires (scale adapted from the TES, Frenzel et al., 2016), cross-sectional design	Preservice secondary school teachers
Rinas et al.	A7. Exploring university instructors' achievement goals and discrete emotions	Discrete teaching emotions: Enjoyment, pride, anger, anxiety, shame, and boredom	Self-report questionnaires (single items adapted from Goetz et al., 2016), cross-sectional design	University instructors
**Section A.3. Focus on Effects**				
Huang et al.	A8. Striving to become a better teacher: linking teacher emotions with informal teacher learning across the teaching career	Discrete teaching emotions: Enjoyment, anger, and anxiety	Self-report questionnaires (TES, Frenzel et al., 2016), cross-sectional design	Elementary school teachers
Rusu and Colomeischi	A9. Positivity ratio and well-being among teachers: The mediating role of work engagement	Positive and negative trait emotions	Self-report questionnaires (PANAS, Watson et al., 1988), cross-sectional design	School teachers
**Section A.4. Focus on reciprocal linkages**				
Frenzel et al.	A10. Who enjoys teaching, and when between-and within-person evidence on teachers' appraisal-emotion links	Discrete teaching emotions: Enjoyment, anger, anxiety	Self-report questionnaires (TES, Frenzel et al., 2016), cross-sectional design	Secondary school teachers
Burić et al.	A11. Teachers' emotions and self-efficacy: A test of reciprocal relations	Discrete teaching emotions: Joy, pride, love, anger, exhaustion and hopelessness	Self-report questionnaires (TEQ, Buric et al., 2018), longitudinal design	School teachers
**PART B: TEACHERS' EMOTION REGULATION AND EMOTIONAL COMPETENCE**				
Aldrup et al.	A12. Measuring teachers' social-emotional competence: development and validation of a Situational Judgment Test	Social-Emotional Competence in terms of teachers' “knowledge, skills, and motivation required to master social and emotional situations,” here specified into emotion regulation capacities and relationship management capacities	Self-report questionnaires (newly developed scales), cross-sectional design	In- and pre-service school teachers
Han et al.	A13. Examining the relationships between job characteristics, emotional regulation and university teachers' well-being: The mediation of emotional regulation	Emotion regulation in terms of cognitive reappraisal vs. expressive suppression	Self-report questionnaires (ERQ, Gross and John, 2003), cross-sectional design	University instructors
Donker et al.	A14. Teachers' emotional exhaustion: Associations with their typical use of and implicit attitudes toward emotion regulation strategies	Emotion regulation in terms of cognitive reappraisal vs. expressive suppression and emotional exhaustion as central aspect of burnout	Implicit attitude assessment for emotion regulation preference (Emotion Regulation-IAT, Mauss et al., 2006) and self-report questionnaires for emotional exhaustion (from MBI, Maslach et al., 1996) and typical emotion regulation strategy use (ERQ, Gross and John, 2003), cross-sectional design	Secondary and vocational teachers, and perservice secondary teachers
Chang	A15. Emotion display rules, emotion regulation, and teacher burnout	Emotion display rules in terms of “principles that guide us to make decisions (…) to express or not to express our emotions,” Emotion regulation in terms of cognitive reappraisal vs. expressive suppression, and Teacher Burnout in terms of the composite of emotional exhaustion, depersonalization, and reduced personal accomplishment	Self-report questionnaires (self-developed scale on display rules, ERQ, Gross and John, 2003, and modified teacher burnout scale by Schaufeli and Salanova, 2007), cross-sectional design	School teachers
Zheng et al.	A16. Leading teachers' emotions like Parents: Relationships between paternalistic leadership, emotional labor and teacher commitment in China	Emotional labor in terms of “the management of feeling to create a publicly observable facial and bodily display”, categorized into deep acting vs. surface acting	Self-report questionnaires (TELSS, Yin et al., 2017), cross-sectional design	Elementary school teachers

Within Part 1 of this collection of papers, which explicitly covers teachers' emotional and affective experiences, the first paper from Chen et al. examines the complexity of teacher emotions by exploring, through computational text analysis, around one million teachers' online posts from 2007 through 2018. The results evidence the multi-dimensionality characteristic of emotions, as the authors identify multiple categories of emotions and various degrees of sentiment polarity of teachers. They also emphasize the dynamic nature of teacher emotions, and report that teacher emotions vary between their workplace and their personal lives, and over time. The second paper, by Chen et al., investigates two highly experienced, award-winning teachers' trajectories of emotional experiences and their emotional labor strategies across their career, using a case-study approach. The findings demonstrate a dynamic pattern of emotions and emotional labor, as teachers transit from one career stage to another. The *third* paper from Cross Francis et al. focuses both on the nature of teacher emotions and on how they are managed in the act of teaching, using qualitative data from seven primary teachers in USA, asking them to report about the emotions they felt in anticipation of teaching, and during teaching retrospectively. The authors identified six emotion categories (positive, negative, neutral, blended-positive, blended-negative, and mixed), while mixed emotions (co-occurrence of positive and negative emotions) are the most dominant. The *fourth* paper (Tsang) takes a sociological perspective, using interview data from 21 teachers at Hong Kong secondary schools as well as policy documents and newspaper articles from the education reform era of 1980–2011, to investigate the social construction of teacher emotions by drawing on the emergent sociological concept/idea of inhabited institutionalism. The author concludes that teachers' emotions can be regarded as an interactional–institutional construction resulting from the negotiation of meaning under the institutional logics and interactions in the context of the school organizations that they inhabit.

Next, Yoo and Rho explore predictors of teacher job satisfaction via the machine learning technique Group Mnet, using data from 2,933 Korean middle school teachers and 177 principals who participated in the OECD 2013 Teaching and Learning International Survey (TALIS). The analysis of this fifth paper identifies 18 predictors of teacher satisfaction, including different school climates, teacher self-efficacy, teacher feedback, and perceived barriers to professional development. The sixth paper by Büssing et al. provides evidence on the topic-specificity of teacher enjoyment, using self-report data from 189 German biology pre-service teachers. The results show that pre-service teachers' anticipated enjoyment of teaching certain topics is differentially predicted by various values, e.g., the value of universalism predicted enjoyment for teaching about the return of wolves, and the value of benevolence predicted enjoyment for teaching about preimplantation genetic diagnosis. The seventh paper by Rinas et al. examines the relationship between achievement goals on discrete emotions, using self-report data from 439 instructors from German and Austrian universities. Results reveal that achievement goals are differentially and meaningfully associated with discrete positive and negative emotions including enjoyment, pride, anger, anxiety, shame, and boredom. For example, learning approach goals are positively related to enjoyment and negatively related to anger and boredom, while learning avoidance goals are positively related to anger.

The eighth paper, a paper by Huang et al. identifies systematic links between teacher enjoyment, anxiety and anger and teachers' engagement in informal teacher learning (learning through media, colleague interaction, stakeholder interaction, student interaction, and individual reflection), using self-report data from 2.880 primary teachers from China. The ninth paper from Rusu and Colomeischi investigates the link between the positive to negative emotion ratio and work engagement and well-being, using self-report data from 1,335 teachers from Romania. The results show teachers with a higher ratio of positive to negative emotions report more engagement and in consequence, higher levels of subjective well-being.

Based on Frenzel (2014) reciprocal model of teacher emotions, the tenth paper by Frenzel et al. identifies the relevance of goal attainment appraisals for teachers' emotions both on a between-person and a within-person level through single- and two-level multivariate multiple regression analyses, using self-report data from 244 German secondary teachers. One of their key results is that teacher–student relationship quality attainment shows particularly strong links with teacher enjoyment, anger, and anxiety, on both levels of analysis. The eleventh paper, by Burić et al. explicitly tests the reciprocal relations between teacher emotions and self-efficacy, using self-report data from 3,010 Croatian teachers in a longitudinal study based on a full panel design with three measurement points and time lags of ~6 months. Results show that the association between teacher emotions and self-efficacy is not bidirectional, but asymmetrical: higher levels of TSE beliefs predict higher levels of positive emotions of joy and pride, while higher levels of teachers' negative emotions of anger, exhaustion, and hopelessness predict lower levels of teachers' self-efficacy beliefs.

Furthermore, in Part 2, this collection of papers contains five papers that address teachers' emotion regulation and emotional competence. The first one of them, paper *twelve*, by Aldrup et al. from Germany is a methodological paper providing validation data for a newly developed situational judgment test measuring teachers' emotion regulation and relationship management capacities (Test of Regulation in and Understanding of Social Situations in Teaching: TRUST), using both in-service and pre-service teacher samples. They provide evidence of the new instrument's internal consistency, as well as its validity as teachers' emotion regulation and relationship management capacity scores show meaningful relations between teachers' emotional intelligence, Big Five personality traits, occupational well-being, and job satisfaction. The thirteenth paper by Han et al. examines the relationships between job characteristics of university teaching, emotion regulation strategies, and well-being using self-report data from 643 university teachers in China. Their results indicate that emotional job demands and teaching support are positively linked with university teachers' use of reappraisal strategies and well-being. In turn, suppression strategies are negatively linked with teachers' well-being. These findings support the mediation role of emotion regulation, and evidence the applicability of the Job Demands-Resources model to a higher education context. The 14 paper by Donker et al. is one of the few papers within this Research Topic which moves beyond pure questionnaire-based self-report by including a computer-based implicit measure of teachers' attitudes toward emotion regulation indicating their preference for regulation over expression, in addition to self-report questionnaires for emotional exhaustion and typical emotion regulation strategy use. Sampling 94 secondary and vocational teachers, as well as preservice secondary teachers from the Netherlands, they find no direct links between teachers' implicit attitude toward emotion regulation and their emotional exhaustion or self-reported strategy use. However, teachers' implicit attitudes toward emotion regulation did moderate the relationship between the use of emotion regulation strategies and emotional exhaustion in the subsample of more experienced teachers. The fifteenth paper of this collection, Chang also addresses teachers' emotion regulation, proposing that it could function as a mediator between emotional labor and burnout as a longer-term emotional outcome. Indeed, this study could show that teachers' display rules are closely positively linked with expressive suppression, which in turn is positively linked with all three dimensions of burnout in a cohort of 561 teachers in the USA. Further, use of cognitive reappraisals is negatively associated with all three dimensions of burnout. Finally, the sixteenth paper of this collection by Zheng et al. also proposes that teacher emotional variables (here: emotional labor in terms of deep vs. surface acting) function as psychological mediators, in this case between principals' leadership behaviors and teachers' commitment. Using self-report data from a sample of 419 primary teachers from China, they could indeed show that deep acting is a positive mediator, and surface acting is a negative mediator between principals' authoritarian vs. benevolent leadership and teachers' commitment both to the profession and to the school.

To sum up, the collection of papers in this Research Topic provides important findings on the complex nature and correlates of teacher emotions. It assembles 16 empirical articles sampled from nine nations namely Australia, Chinese mainland, Croatia, Germany, Hong Kong, Korea, Romania, the Netherlands, and the USA. It thus represents a truly international and inter-cultural mix of data sources and perspectives on the topic. The articles cover various, mostly impressively large samples, from primary to tertiary levels. Moreover, this collection grounds a wide range of theoretical approaches, conceptual frameworks and methods of inquiry, which allows for highly diverse and nuanced findings regarding teacher emotions as well as related emotional variables, specifically teacher emotion regulation. We hope that the contributions from this Research Topic will spark further scientific scholarly work on the topic, and inspire and serve policymakers and practitioners.

## Author Contributions

All authors listed have made a substantial, direct and intellectual contribution to the work, and approved it for publication.

## Conflict of Interest

The authors declare that the research was conducted in the absence of any commercial or financial relationships that could be construed as a potential conflict of interest.

## Footnotes

^1^The search terms used were teach* AND Emotion* OR feeling* OR affect* OR mood* in the title, keywords, or abstract of the journal articles during the period between 1985 and 2019. The databases included ERIC, ProQuest, PsycArticles, PsycINFO, SAGE, ScienceDirect, Scopus, and Web of Science.

## References

[B1] Buric I. Sliskovic A. Macuka I. (2018). A mixed-method approach to the assessment of teachers' emotion: development and validation of the Teacher Emotion Questionnaire. Educ. Psychol. 38, 325–349. 10.1080/01443410.2017.1382682

[B2] Chen J. (2019). Research review on teacher emotion in Asia between 1988 and 2017: Research Topics, research types, and research methods. Front Psychol. 10:1628. 10.3389/fpsyg.2019.01628 31496964 PMC6712823

[B3] Chen J. (2020). Refining the teacher emotion model: evidence from a review of literature published between 1985 and 2019. Cambr. J. Educ. 10.1080/0305764X.2020.1831440

[B4] Frenzel A.C. (2014). Teacher emotions, in International Handbook of Emotions in Education, eds Linnenbrink-Garcia E. A. Pekrun R. New York, NY: Routledge, 494–519.

[B5] Frenzel A. C. Pekrun R. Gostz T. Daniels L. M. Durksen T. L. Becker-Kurz B.. (2016). Measuring Teachers' enjoyment, anger, and anxiety: the Teacher Emotions Scales (TES). Contempo. Educ. Psychol. 46, 148–163. 10.1016/j.cedpsych.2016.05.00326053623

[B6] Fried L. Mansfield C. Dobozy E. (2015). Teacher emotion research: Introducing a conceptual model to guide future research. Issues Educ. Res. 25, 415–441.

[B7] Goetz T. Bieg M. Hall N. C. (2016). Assessing academic emotions via the experience sampling method, in Methodological Advances in Research on Emotion and Education, eds Zembylas M. Schutz P. A. (Cham: Springer), 245–258. 10.1007/978-3-319-29049-2_19

[B8] Gross J. J. John O. P. (2003). Individual differences in two emotion regulation processes: implications for affect, relationships, and well-being. J. Person. Social Psychol. 85, 348–362. 10.1037/0022-3514.85.2.348 12916575

[B9] Maslach C. Jackson S. E. Leiter M. P. (1996). Maslach Burnout Inventory manual. 3th Edn. Palo Alto, CA: Consulting Psychologists Press.

[B10] Mauss I. B. Evers C. Wilhelm F. H. Gross J. J. (2006). How to bite your tongue without blowing your top: implicit evaluation of emotion regulation predicts affective responding to anger provocation. Pers. Soc. Psychol. Bull. 32, 589–602. 10.1177/0146167205283841 16702153

[B11] Nias J. (1996). Thinking about feeling: the emotions in teaching. Camb. J. Educ. 26, 293–306. 10.1080/0305764960260301

[B12] Schaufeli W. B. Salanova M. (2007). Work engagement: an emerging psychological con-cept and its implications for organizations, in Managing Social and Ethical Issues in Organizations, eds Gilliland S. W. Steiner D. D. Skarlicki D. P., Vol. 5 (Greenwich, CT: Information Age Publishers), 135–177.

[B13] Uitto M. Jokikokko K. Estola E. (2015). Virtual special issue on teachers and emotions in Teaching and Teacher Education (TATE) in 1985-2014. Teach. Teach. Educ. 50, 124–135. 10.1016/j.tate.2015.05.008

[B14] Watson D. Clark L. A. Tellegen A. (1988). Development and validation of brief measures of positive and negative affect: the PANAS scales. J. Person. Social Psychol. 54, 1063–1070. 10.1037/0022-3514.54.6.1063 3397865

[B15] Yin H. Huang S. Chen G. (2019). The relationships between teachers' emotional labor and their burnout and satisfaction: a meta-analytic review. Educ. Res. Rev. 28:100283 10.1016/j.edurev.2019.100283

[B16] Yin H. Huang S. Lee J. C. K. (2017). Choose your strategy wisely: examining the relationships between emotional labor in teaching and teacher efficacy in Hong Kong primary schools. Teach. Teach. Educ. 66, 127–136. 10.1016/j.tate.2017.04.006

